# Fokus neurologische Intensivmedizin. Intensive Care Studies from 2020/2021

**DOI:** 10.1007/s00101-021-00977-w

**Published:** 2021-06-30

**Authors:** D. Michalski, C. Jungk, T. Brenner, M. Dietrich, C. Nusshag, C. J. Reuß, M. O. Fiedler, M. Bernhard, C. Beynon, M. A. Weigand

**Affiliations:** 1 Klinik und Poliklinik für Neurologie, Universitätsklinikum Leipzig, Liebigstraße 20, 04103 Leipzig, Deutschland; 2 Neurochirurgische Klinik, Universitätsklinikum Heidelberg, Heidelberg, Deutschland; 3 Klinik für Anästhesiologie und Intensivmedizin, Universitätsklinikum Essen, Essen, Deutschland; 4 Klinik für Anästhesiologie, Universitätsklinikum Heidelberg, Heidelberg, Deutschland; 5 Klinik für Endokrinologie, Stoffwechsel und klinische Chemie/Sektion Nephrologie, Universitätsklinikum Heidelberg, Heidelberg, Deutschland; 6 Klinik für Anästhesiologie und operative Intensivmedizin, Klinikum Stuttgart, Stuttgart, Deutschland; 7 Zentrale Notaufnahme, Universitätsklinikum Düsseldorf, Heinrich-Heine-Universität, Düsseldorf, Deutschland

## Übersicht aller 5 Artikel zu den intensivmedizinische Studien aus 2020/2021

- Fokus allgemeine Intensivmedizin: www.springermedizin.de/link/10.1007/s00101-021-00976-x
- Fokus neurologische Intensivmedizin: www.springermedizin.de/link/10.1007/s00101-021-00977-w
- Fokus neurochirurgische Intensivmedizin: www.springermedizin.de/link/10.1007/s00101-021-00978-9
- Fokus Beatmung: www.springermedizin.de/link/10.1007/s00101-021-00979-8
- Fokus Nephrologie: www.springermedizin.de/link/10.1007/s00101-021-00980-1

Die Beiträge stehen Ihnen sofort nach der Fertigstellung auf www.springermedizin.de zur Verfügung. Bitte geben Sie dort den Beitragstitel in die Suche ein.

## Lokalanästhesie bei der mechanischen Thrombektomie

### Originalpublikation

Benvegnù F, Richard S, Marnat G et al (2020) Local anesthesia without sedation during thrombectomy for anterior circulation stroke is associated with worse outcome. Stroke 51:2951–2959.

Untersuchungen zum optimalen periprozeduralen Management der mechanischen Thrombektomie fokussierten in den vergangenen Jahren v. a. auf die Verfahren der Allgemeinanästhesie und Analgosedierung. Untersuchungen auf der Basis retrospektiver Betrachtungen hatten hierzu einen Vorteil für die Sedierung geliefert [5], wohingegen die zu diesem Thema zuletzt veröffentlichte, ausschließlich prospektive Studien einbeziehende Metaanalyse einen Vorteil für die protokollbasierte Allgemeinanästhesie in Bezug auf das funktionelle Behandlungsergebnis nach 3 Monaten ergeben hatte [24].

Benvegnù et al. [3] untersuchten nun die Effektivität und die Sicherheit einer ausschließlichen Lokalanästhesie (ca. 10 ml Lidocain s.c.) im Bereich der Punktionsstelle als periprozedurales Management während der mechanischen Thrombektomie im Vergleich zur Analgosedierung mit i.v.-Gabe von Remifentanil. Hierfür wurden Daten aus einem prospektiv angelegten Register verwendet, in das 4 Zentren in Frankreich im Zeitraum von Januar bis Dezember 2018 insgesamt 1034 Patienten mit einem proximalen Gefäßverschluss eingeschlossen hatten. Von diesen erhielten 762 Patienten als primären Behandlungsstandard in 3 Zentren eine Analgosedierung und 272 Patienten als primären Behandlungsstandard in einem Zentrum eine Lokalanästhesie. Nach Ausschluss von Fällen, bei denen sich im Verlauf der mechanischen Thrombektomie eine Änderung des Verfahrens ergeben hatte bzw. Daten fehlten, verblieben 222 Patientenpaare in der „Intention-to-treat“-Analyse. Primärer Endpunkt war ein gutes Behandlungsergebnis, definiert als ein Wert von 0 bis 2 auf der modifizierten Rankin-Skala (mRS) nach 3 Monaten. Dieser wurde bei 89 der 222 Patienten (40 %) mit erfolgter Lokalanästhesie und bei 116 der 222 Patienten (52 %) mit erfolgter Analgosedierung erreicht, sodass ein signifikanter Vorteil für die Analgosedierung erkennbar wurde (*p* = 0,028). Dagegen unterschieden sich die Raten von symptomatischen intrakraniellen Blutungen (9,9 vs. 8,3 %; *p* = 0,58) und prozeduralen Komplikationen (10,2 vs. 8,5 %; *p* = 0,59) nicht zwischen der Lokalanästhesie und der Analgosedierung; auch die Sterblichkeit innerhalb von 90 Tagen war vergleichbar (24,3 vs. 18,2; *p* = 0,17). Hinsichtlich des prozeduralen Erfolges war eine Reperfusion („modified treatment in cerebral infarction“ [mTICI] 2b-3) bei 170 der 220 Patienten (76,6 %) mit erfolgter Lokalanästhesie und bei 193 der 220 Patienten (87,1 %) mit erfolgter Analgosedierung erreicht worden, sodass sich ein signifikanter Vorteil für die Analgosedierung ergab (*p* = 0,012).

Die Studie von Benvegnù et al. [3] liefert anhand einer relativ umfangreichen Stichprobe einen statistischen Vorteil für eine Analgosedierung mit Remifentanil während der mechanischen Thrombektomie gegenüber der reinen Lokalanästhesie. Hierbei zu berücksichtigen ist der Erhebungszeitraum, der mit dem Jahr 2018 deutlich nach dem Einführungszeitraum der mechanischen Thrombektomie um das Jahr 2015 liegt. Bereits gut etablierte Prozeduren im Zusammenhang mit der Analgosedierung sind für die vor Ort tätigen neuroradiologischen und anästhesiologischen Teams anzunehmen, sodass die Lokalanästhesie hier als neueres und möglicherweise noch nicht vertrautes Verfahren zu bewerten ist. Die mögliche Bedeutung standardisierter Protokolle wird durch die Autoren selbst insofern hervorgehoben, als dass beispielsweise die Vermeidung einer Hypotonie fester Bestandteil des Protokolls für die Analgosedierung in den durchführenden Zentren war, dies jedoch in der Gruppe der Lokalanästhesie nicht als Ziel definiert war [3]. Auch unterschieden sich die beiden Gruppen hinsichtlich der Anwesenheit eines Anästhesisten, die lediglich im Falle der Analgosedierung gegeben war. Nachdem eine frühere, retrospektive Untersuchung anhand einer kleineren Stichprobe den Hinweis für einen Vorteil der Lokalanästhesie ergeben hatte [28], erscheinen analog zur Beurteilung der Effekte der Allgemeinanästhesie auch beim Vergleich der Analgosedierung mit der Lokalanästhesie Faktoren wie das Studiendesign und die vor Ort genutzten Behandlungsprotokolle eine relevante Bedeutung zu haben. Prospektive randomisierte Studien mit gut strukturierten Protokollen sind daher notwendig, um die Vor- und Nachteile der Lokalanästhesie gegenüber der Analgosedierung offenzulegen. Erst hieraus werden sich Implikationen für die Praxis ableiten lassen.

## Blutdruckmanagement während und nach der mechanischen Thrombektomie

### Originalpublikation

Fandler-Höfler S, Heschl S, Argüelles-Delgado P et al (2020) Single mean arterial blood pressure drops during stroke thrombectomy under general anaesthesia are associated with poor outcome. J Neurol 267:1331–1339.

Ausgehend von der Befürchtung einer im Rahmen der Allgemeinanästhesie auftretenden Hypotonie mit neurologischer Verschlechterung infolge einer zerebralen Hypoperfusion fokussierten retrospektive Studien frühzeitig auf den Blutdruck während der mechanischen Thrombektomie. Die Ergebnisse waren dabei wenig konklusiv, da einerseits kein statistischer Zusammenhang zwischen Blutdruckabfällen während der Prozedur und dem längerfristigen Behandlungsergebnis nachvollziehbar war [25], andere Studien dagegen erhöhte Blutdruckwerte [15] ebenso wie erniedrige Blutdruckwerte [22] mit einem schlechteren Behandlungsergebnis in Verbindung brachten. Zu den methodischen Einschränkungen dieser Studien gehörte die Berücksichtigung punktueller Blutdruckwerte wie beispielsweise unmittelbar vor und nach der Rekanalisation des Gefäßes.

Fandler-Höfler et al. [12] untersuchten nun in einer retrospektiven, monozentrischen Kohortenstudie den Zusammenhang von periprozedural erhobenen Blutdruckwerten und dem funktionellen Behandlungsergebnis nach 3 Monaten. Ein entscheidendes Merkmal der Studie war die Einbeziehung von Blutdruckdaten, die aus einer kontinuierlichen invasiven Messung während der in Allgemeinanästhesie stattfindenden Prozedur stammten. Verwendet wurden die Daten von 115 Patienten, die im Zeitraum 2011 bis 2016 in Graz (Österreich) mit einer mechanischen Thrombektomie aufgrund eines proximalen Gefäßverschlusses behandelt wurden. Von diesen erzielten 59 Patienten (51,3 %) ein gutes und 56 Patienten (48,7 %) ein ungünstiges Behandlungsergebnis nach 3 Monaten, definiert anhand der mRS (0–2 vs. 3–6). Der über die gesamte Prozedur gemittelte systolische (*p* = 0,54), diastolische (*p* = 0,75) und auch der mittlere arterielle Druck (*p* = 0,73) unterschieden sich nicht zwischen den vorgenannten beiden Gruppen. Ein zu irgendeinem Zeitpunkt auftretender Blutdruckabfall auf einen mittleren arteriellen Blutdruck von weniger als 60 mm Hg war dagegen mit einem ungünstigen funktionellen Ergebnis (mRS 3–6) assoziiert (*p* = 0,01), was sich in einer Shift-Analyse zum mRS (*p* < 0,01) und auch in einer multivariaten Analyse unter Einbeziehung des Patientenalters und vorbestehender Erkrankungen wie einer arteriellen Hypertonie und eines Diabetes mellitus bestätigte (*p* < 0,01).

Die Studie von Fandler-Höfler et al. [12] bestätigt die Bedeutung eines optimalen periprozeduralen Blutdruckmanagements, wobei nach dem aktuellen Wissensstand Blutdruckabfälle vermieden werden sollen. Weil sich diese Ergebnisse auf die Allgemeinanästhesie beziehen, sind weiterführende Studien zum optimalen Blutdruckbereich in Abhängigkeit von der individuell gewählten Form des periprozeduralen Managements (Allgemeinanästhesie, Analgosedierung und reine Lokalanästhesie) notwendig.

### Originalpublikation

Mazighi M, Richard S, Lapergue B et al (2021) Safety and efficacy of intensive blood pressure lowering after successful endovascular therapy in acute ischaemic stroke (BP-TARGET): a multicentre, open-label, randomised controlled trial. Lancet Neurol, im Druck.

Hinsichtlich des optimalen Blutdruckmanagements nach der mechanischen Thrombektomie lieferten retrospektive Untersuchungen bereits einen statistischen Zusammenhang zwischen der Höhe des Blutdrucks sowie dem funktionellen Behandlungsergebnis und dem Auftreten von Blutungskomplikationen [15], wobei sich als systolische Obergrenze ein Wert von 140 mm Hg [2] bzw. 160 mm Hg [16] abzeichnete.

In einer randomisierten kontrollierten Studie mit der markanten Bezeichnung BP-TARGET, untersuchten Mazighi et al. [17] nun den Effekt einer strikten Blutdruckkontrolle (systolisch 100–129 mm Hg) gegenüber der Standardbehandlung (systolisch 130–185 mm Hg), wobei die Zielbereiche innerhalb 1 h nach der Randomisierung erreicht und für 24 h aufrechterhalten werden mussten. Zwischen 2017 und 2019 wurden in 4 Zentren in Frankreich insgesamt 324 Patienten mit einem Verschluss der A. cerebri media im M1-Segment oder der A. carotis interna im distalen Abschnitt eingeschlossen. Sechs Patienten mussten u. a. aufgrund zurückgezogener Einwilligungen ausgeschlossen werden. Es verblieben 158 Patienten in der Gruppe der strikten Blutdruckkontrolle und 160 in der Gruppe der Standardtherapie. Primärer Endpunkt war die Rate an intraparenchymatösen Blutungen, beurteilt in der radiologischen Diagnostik nach 24–36 h. Diese traten in Verbindung mit einer strikten Blutdruckkontrolle bei 67 von 158 Patienten (42 %) und im Zusammenhang mit der Standardbehandlung bei 69 der 160 Patienten (43 %) auf. Als Sicherheitsendpunkt wurde zudem das Auftreten einer Hypotension (systolischer Blutdruck < 80 mm Hg) berücksichtigt, die in der Gruppe mit strikter Blutdruckkontrolle bei 12 Patienten (8 %) und in der Gruppe mit Standardbehandlung bei 5 Patienten (3 %) vorkam. Erfasst wurde auch die Rate an Patienten mit einer neurologischen Verschlechterung, definiert als eine Zunahme der National Institutes of Health Stroke Scale von mehr als 4 Punkten, die in der gesamten Population lediglich bei einem Patienten (< 1 %) auftrat und im Zusammenhang mit der strikten Blutdruckkontrolle stand. Auch die Anzahl der Patienten mit einem guten Behandlungsergebnis nach 3 Monaten, definiert als ein Score von 0–2 auf der mRS, unterschied sich nicht zwischen der Gruppe mit strikter Blutdruckkontrolle (67 von 152 Patienten, 44 %) und der Standardbehandlung (69 von 153 Patienten, 45 %).

Die Studie von Mazighi et al. [17] liefert damit auf den ersten Blick keinen Vorteil einer strikten Blutdruckkontrolle innerhalb der ersten 24 h nach einer mechanischen Rekanalisation. Die Vorteile der Studie liegen in dem randomisierten Design und der Auswahl funktionell relevanter Endpunkte (Blutungen als primärer Endpunkt, funktionelles Behandlungsergebnis als sekundärer Endpunkt). Eine mögliche Begründung für das unerwartete Ergebnis der Studie könnte in den unterschiedlich gewählten Umfängen der systolischen Blutdruckbereiche liegen (100–129 vs. 130–185 mm Hg). Der Begriff Standardbehandlung erscheint sogar irreführend, weil sich die damit verbundene Obergrenze von 185 mm Hg an den Empfehlungen der systemischen Thrombolyse orientiert, die nicht die mechanische Thrombektomie als ein in der Regel additives Behandlungselement berücksichtigen. Der direkte Vergleich der innerhalb der ersten 24 h erreichten systolischen Blutdruckwerte zwischen der Gruppe mit strikter Blutdruckkontrolle und der Standardbehandlung ergibt mit 128 vs. 138 mm Hg keinen nennenswerten Unterschied. Zudem bewegt sich der erzielte Blutdruckwert in der Gruppe der Standardbehandlung mit 138 mm Hg unterhalb der aus retrospektiven Studien abgeleiteten Obergrenzen von 140 [2] und 160 mm Hg [16] für die mechanische Thrombektomie. Ein echter Vergleich zwischen diesen Grenzwerten und einem Blutdruckbereich bis 185 mm Hg kann somit der Untersuchung von Mazighi et al. [17] nicht entnommen werden. Weitere randomisierte Untersuchungen unter Nutzungen begrenzterer Blutdruckbereiche wären somit wünschenswert. Die vielerorts bereits angewandte Praxis einer strengeren Blutdruckkontrolle nach der mechanischen Rekanalisation könnte diese jedoch deutlich erschweren.

## Hemikraniektomie nach mechanischer Rekanalisation

### Originalpublikation

Alzayiani M, Schmidt T, Veldeman M et al (2021) Risk profile of decompressive hemicraniectomy for malignant stroke after revascularization treatment. J Neurol Sci 420:117275.

Dass die mechanische Thrombektomie im Vergleich zur alleinigen systemischen Thrombolyse oder auch der konservativen Therapie bei Patienten mit proximalem Gefäßverschluss nachhaltig positive Effekte aufweist, konnte in großen Metaanalysen gezeigt werden [14]. Damit einher gingen auch Veränderungen in der Behandlungslandschaft des Hirninfarkts. So verringerte sich die Auftretenswahrscheinlichkeit eines raumfordernden (malignen) Hirninfarkts signifikant durch die mechanische Thrombektomie [13], was vielerorts zu einer spürbaren Abnahme der Häufigkeit dieses Krankheitsbildes führt. Trotz dieser Fortschritte existiert im Alltag durchaus die Behandlungssequenz einer frühzeitigen und effektiven mechanischen Thrombektomie mit im weiteren Verlauf auftretender raumfordernder Infarktentwicklung. Bei der Indikationsstellung für eine dekompressive Hemikraniektomie treten während der individuellen Nutzen-Risiko-Abwägung Unsicherheiten hinsichtlich der Sicherheit eines operativen Eingriffs nach einer vorangegangenen rekanalisierenden Therapie auf. Hintergrund ist ein möglicherweise erhöhtes Blutungsrisiko durch das Zusammentreffen von Reperfusion und einem operativen Eingriff.

Mithilfe einer retrospektiven Kohortenstudie untersuchten Alzayiani et al. [1] nun die Auswirkungen einer rekanalisierenden Therapie auf die nachfolgende Hemikraniektomie. Berücksichtigt wurden 152 Patienten, die zwischen 2012 und 2015 in Aachen mit einer dekompressiven Hemikraniektomie behandelt wurden. Ausgeschlossen wurden Patienten mit der vorherigen Einnahme von Thrombozytenaggregationshemmern oder Antikoagulanzien, da dies das Auftreten perioperativer Blutungskomplikationen in relevantem Maße mitbedingen könnte. Von den verbleibenden 50 Patienten erhielten 24 (48 %) vor der Hemikraniektomie eine rekanalisierende Therapie. Im Detail erfolgten bei 14 Patienten ausschließlich eine systemische Thrombolyse, bei 4 Patienten ausschließlich eine mechanische Thrombektomie und bei 7 Patienten eine Kombination dieser Verfahren. Im Ergebnis unterschieden sich Patienten mit einer dekompressiven Hemikraniektomie und vorangegangener Rekanalisation gegenüber operierten Patienten ohne vorangegangene Rekanalisation nicht hinsichtlich der Operationsdauer (83 vs. 96 min; *p* = 0,308), dem intraoperativen Blutverlust (300 vs. 300 ml; *p* = 0,763) und der intraoperativen Transfusionspflichtigkeit (12,5 vs. 26,9 %; *p* = 0,294). Im postoperativen Verlauf fanden sich ebenso keine Unterschiede hinsichtlich Blutungskomplikationen (16,7 vs. 19,5 %; *p* = 0,814), notwendiger Revisionen infolge von Blutungen (0 vs. 11,1 %; *p* = 0,697) oder Infektionen (12,5 vs. 7,7 %; *p* = 0,697) und der Sterblichkeit (12,5 vs. 15,4; *p* = 0,769).

In dieser, vermutlich ersten Untersuchung potenzieller Effekte der rekanalisierenden Therapie auf eine nachfolgende dekompressive Hemikraniektomie fanden Alzayiani et al. [1] kein vermehrtes Auftreten von unmittelbaren Komplikationen und dabei insbesondere Blutungen, wie sie infolge einer Vulnerabilitätszunahme unter den Bedingungen der Reperfusion denkbar wären. Wenngleich eine Verallgemeinerung mit dieser Studie nicht möglich ist, gibt sie doch Grund zur Annahme, dass im Nachgang der medikamentösen bzw. mechanischen Rekanalisation ein besonderes Risiko für unmittelbare Komplikationen der Hemikraniektomie nicht besteht, was deren Indikationsstellung erleichtert. Da ein relevanter Anteil der Schlaganfallpatienten eine vorherige gerinnungsmodifizierende Therapie in Form von Thrombozytenaggregationshemmern oder einer Antikoagulation aufweist, wären Untersuchungen zum Risikoprofil speziell dieser Patienten von Interesse.

## Bakterielle Meningitis

### Originalpublikation

Tubiana S, Varon E, Biron C et al (2020) Community-acquired bacterial meningitis in adults: in-hospital prognosis, long-term disability and determinants of outcome in a multicentre prospective cohort. Clin Microbiol Infect 26:1192–1200.

Wenngleich in den vergangenen Jahren eine Reduktion der Inzidenz ambulant erworbener bakterieller Meningitiden in Industrieländern zu verzeichnen war [7], handelt es sich nach wie vor um ein kritisches und mit einer hohen Sterblichkeit verbundenes Krankheitsbild. Das Erregerspektrum ist vom Patientenalter abhängig, wobei in der älteren Bevölkerung als häufigste Erreger *Streptococcus pneumoniae, Listeria monocytogenes* und *Neisseria meningitidis* zu finden sind [6]. Zu den Therapiestrategien gehören die schnellstmögliche und adäquate antibiotische Behandlung, unter bestimmten Bedingungen zusammen mit der Gabe von Dexamethason, und in jedem Fall die Fokussuche [20]. Aufgrund der üblicherweise geringen Fallzahlen pro Behandlungszentrum sind Untersuchungen zu den Faktoren, die mit einem ungünstigen Verlauf einhergehen, naturgemäß schwierig.

In einer prospektiven Kohortenstudie untersuchten Tubiana et al. [27] nun 533 Patienten, die in 69 Zentren in Frankreich mit einer ambulant erworbenen bakteriellen Meningitis behandelt wurden, hinsichtlich zahlreicher Faktoren und des Langzeitverlaufs bis ein Jahr nach der Erkrankung. Als häufigster Erreger konnte *Streptococcus pneumoniae* eingegrenzt werden, der in 53,8 % der Fälle nachzuweisen war. Neunzig der 533 Patienten (16,9 %) verstarben bereits während des Krankenhausaufenthalts, und bei immerhin 225 von 500 Patienten (45 %) lag ein ungünstiges Behandlungsergebnis (Score 2–6 auf der mRS) am Ende des Krankenhausaufenthalts vor. Mit diesem assoziiert waren u. a. ein Patientenalter von mehr als 70 Jahren (*p* = 0,0054), ein männliches Geschlecht, fokale neurologische Symptome (*p* < 0,0001), eine chronische Niereninsuffizienz (*p* = 0,0100), eine disseminierte intravasale Gerinnung (*p* = 0,0250) sowie eine verzögerte, d. h. eine erst nach mehr als einem Tag stattfindende Lumbalpunktion (*p* = 0,0083) und liquorspezifische Parameter. Hinsichtlich der Liquoruntersuchungen waren erwartungsgemäß eine sehr niedrige Glucosekonzentration (*p* = 0,0482) und ein erhöhter Proteingehalt (*p* = 0,0066), jedoch auch eine Leukozytenzahl von weniger als 1500/µl (*p* = 0,0010) mit einem ungünstigen Behandlungsergebnis assoziiert. Im Nachbeobachtungszeitraum fanden sich bei 74 von 277 Patienten (26,7 %) ein Hörverlust, bei 86 von 277 Patienten (31 %) persistierende Kopfschmerzen sowie bei 87 von 265 Patienten (32,8 %) depressive Symptome und bei 142 von 266 Patienten (53,4 %) Einschränkungen der gesundheitsbezogenen Lebensqualität.

Die Studie von Tubiana et al. [27] besticht v. a. durch die vergleichsweise große, nur durch den Zusammenschluss mehrerer Behandlungszentren möglich gewordene Stichprobengröße. Durch die gefundenen Assoziationen einzelner Faktoren mit einem ungünstigen Behandlungsergebnis kann die Studie eine Hilfestellung bei der frühzeitigen Identifizierung ungünstiger Verläufe geben. Frühere Untersuchungen bestätigend, war eine verzögerte Diagnosestellung mit einem ungünstigen Behandlungsergebnis assoziiert, was den Faktor Zeit in der initialen Diagnostik noch einmal unterstreicht. Der relevante Anteil an Patienten mit Einschränkungen im Langzeitverlauf könnte als Ausgangspunkt für spezifische Nachsorgekonzepte genutzt werden.

## COVID-19 und Schlaganfall

### Originalpublikation

Qureshi AI, Baskett WI, Huang W et al (2021) Acute ischemic stroke and COVID-19: an analysis of 27,676 patients. Stroke 52:905–912.

Ausgehend von Fallberichten wurde als mögliche neurologische Komplikation einer Infektion mit SARS-CoV‑2 frühzeitig im Verlauf der Pandemie der ischämische Schlaganfall diskutiert [23]. Möglicherweise aus Gründen einer veränderten Ressourcenverteilung war jedoch weltweit ein Rückgang der infolge eines Schlaganfalls hospitalisierten Patienten zu beobachten, sodass die Herstellung kausaler Beziehungen offenblieb [4]. Erste gepoolte Analysen von Patienten mit einem ischämischen Schlaganfall und dem laborgestützten Nachweis von SARS-CoV‑2 legten schwerere Verläufe, ein ungünstigeres funktionelles Behandlungsergebnis und eine erhöhte Sterblichkeit nahe [19].

In einer umfangreichen retrospektiven Analyse von Qureshi et al. [21] wurden nun Risikofaktoren, Komorbiditäten, Behandlungsstrategien und -ergebnisse von Patienten mit einem ischämischen Schlaganfall aus einer Kohorte von einerseits klinischen Verdachtsfällen ohne bestätigenden Nachweis und andererseits laborchemisch bestätigten Erkrankungen durch SARS-CoV‑2 („coronavirus disease 2019“, COVID-19) anhand von ICD-Codes untersucht. Zugrunde lag eine Datenbank („Cerner De-Identified COVID-19 Dataset“ als Teil der „Cerner Real-World Data“), in der Informationen von 27.676 Patienten vorlagen, eingepflegt von 54 US-amerikanischen Zentren in den Jahren 2019 und 2020. Im Ergebnis konnten 8163 Patienten mit COVID-19-Erkrankung identifiziert werden, von denen 103 Patienten (1,3 %) einen Schlaganfall entwickelten. Von den verbleibenden 19.513 Patienten mit initial zwar bestehendem klinischem Verdacht, letztlich jedoch fehlender COVID-19-Erkrankung, entwickelten 199 Patienten (1,0 %) einen Schlaganfall.

COVID-19-Patienten mit einem Schlaganfall unterschieden sich von denen ohne Schlaganfall durch ein höheres mittleres Alter (68,8 vs. 54,4 Jahre; *p* < 0,0001) sowie durch das häufigere Vorliegen einer arteriellen Hypertonie (84,5 vs. 48,2 %; *p* < 0,0001), eines Diabetes mellitus (56,3 vs. 30,2 %; *p* < 0,0001), einer Hyperlipidämie (75,7 vs. 33,3 %; *p* < 0,0001) und eines Vorhofflimmerns (28,2 vs. 10,1 %; *p* < 0,0001). Ebenso waren in der Gruppe der COVID-19-Patienten mit einem Schlaganfall signifikant mehr Komplikationen wie ein zerebrales Ödem (13,9 vs. 0,3 %; *p* < 0,0001), ein respiratorisches Versagen (52,4 vs. 29,6 %; *p* < 0,0001) und ein akutes Nierenversagen (50,5 vs. 22,8 %; *p* < 0,0001) nachzuvollziehen. Unterschiede im Auftreten einer tiefen Venenthrombose fanden sich dagegen nicht. Die Sterblichkeit während des Krankenhausaufenthalts war in der Gruppe der COVID-19-Patienten mit einem Schlaganfall gegenüber COVID-19-Patienten ohne Schlaganfall signifikant erhöht (19,4 vs. 6,2 %; *p* < 0,0001) und bezogen auf die poststationäre Versorgungsform konnten signifikant weniger Patienten (29,1 vs. 62,1 %; *p* < 0,0001) in das häusliche Umfeld entlassen werden.

Schlaganfallpatienten mit COVID-19 unterschieden sich von Schlaganfallpatienten ohne COVID-19 dagegen nicht hinsichtlich üblicher Risikofaktoren wie einer arteriellen Hypertonie, eines Diabetes mellitus, einer Hyperlipidämie und eines Vorhofflimmerns. Auch fanden sich keine Unterschiede im Auftreten von Komplikationen wie die Ausbildung eines zerebralen Ödems, eines respiratorischen Versagens, eines akuten Nierenversagen und einer tiefen Venenthrombose. Unterschiede fanden sich ebenso nicht hinsichtlich der Akutbehandlung in Form der systemischen Thrombolyse (1,0 vs. 1,0 %; *p* = 0,98) und mechanischen Thrombektomie (1,0 vs. 1,0 %; *p* = 0,98) sowie der Sterblichkeit innerhalb des Krankenhausaufenthalts (19,4 vs. 21,6 %; *p* = 0,66). Jedoch war die Rate an Krankenhausentlassungen in das häusliche Umfeld bei Schlaganfallpatienten mit COVID-19 signifikant geringer (48,2 %) gegenüber Schlaganfallpatienten ohne COVID-19 (61,1 %) (*p* = 0,02).

In einer bemerkenswert großen Stichprobe fanden Qureshi et al. [21] keinen Unterschied in der Häufigkeit von Schlaganfällen zwischen Patienten mit COVID-19 und denen mit letztlich nichtbestätigter Erkrankung. In der Gruppe der Patienten mit COVID-19 war das Auftreten eines Schlaganfalls mit den üblichen vaskulären Risikofaktoren, aber auch mit einem 2‑fach erhöhten Risiko für ein Versterben innerhalb des Krankenhausaufenthalts und eine Entlassung in das nichthäusliche Umfeld assoziiert. Die Studie liefert damit einen wichtigen Baustein in dem sich ständig erweiternden Wissensschatz um mögliche Zusammenhänge zwischen einer Infektion mit SARS-CoV‑2 und dem ischämischen Schlaganfall. Die in der Studie von Qureshi et al. [21] im Zusammenhang mit COVID-19 und einem Schlaganfall vermehrt wahrgenommenen Organdysfunktionen könnten als Begründung für das relevant schlechtere funktionelle Behandlungsergebnis und die erhöhte Sterblichkeit in dieser Risikogruppe herangezogen werden. Bemerkenswert ist ferner die mit einem Prozent sehr geringe Rate an erfolgter Akuttherapie, für die als Gründe verkomplizierte Abläufe bei der mechanischen Thrombektomie sowie eine relevant verzögerte Krankenhauseinweisung oder eine Verzögerung in der eigeninitiierten Vorstellung unter Pandemiebedingungen naheliegend wären. Diesbezüglich scheinen jedoch regionale Unterschiede zu existieren, da eine kürzliche Untersuchung keine Reduktion der systemischen Thrombolysen und mechanischen Thrombektomien im Berliner Raum unter Pandemiebedingungen zeigen konnte [11].

## Delir

### Originalpublikation

Smit L, Dijkstra-Kersten SMA, Zaal IJ et al (2021) Haloperidol, clonidine and resolution of delirium in critically ill patients: a prospective cohort study. Intensive Care Med 47:316–324.

Entsprechend früherer Untersuchungen tritt bei kritisch kranken, beatmungspflichtigen Patienten in etwa der Hälfte der Fälle ein Delir auf, das statistisch mit einer verlängerten Beatmungszeit und einem verlängerten stationären Aufenthalt assoziiert ist [8, 18]. Dieser enormen Bedeutung im intensivmedizinischen Setting stehen fortbestehende Unsicherheiten in der optimalen medikamentösen Therapie gegenüber, wobei die einschlägige Leitlinie eine Therapie mit niedrig dosierten Neuroleptika und α_2_-Agonisten als geeignet betrachtet [9, 10].

Smit et al. [26] untersuchten nun mit einer prospektiven Kohortenstudie die Auswirkungen einer Behandlung mit dem Neuroleptikum Haloperidol und dem α_2_-Agonisten Clonidin auf den Verlauf des Delirs. Eingeschlossen wurden 3614 der insgesamt 12.380 auf einer gemischten Intensivstation der Universität Utrecht (Niederlande) in den Zeiträumen 2011 und 2013 sowie 2015 und 2019 behandelten Patienten. Auf Tagesebene wurden für die Definition eines Delirs herangezogen: „Richmond Agitation Sedation Scale“ (RASS), „Confusion Assessment Method for Intensive Care Unit“ (CAM-ICU), zusätzliche klinische Zeichen des Delirs und Untersuchungen durch trainiertes Personal. Ausgeschlossen wurden u. a. Patienten mit einem Intensivaufenthalt von weniger als 24 h sowie akute neurologische und neurochirurgische Krankheitsbilder. Innerhalb der untersuchten Kohorte mit insgesamt 24.906 Behandlungstagen fanden sich 4708 Tage, an denen ein Delir vorlag (18,9 %). Bei 1101 Patienten (23,4 %) war im Zusammenhang mit der Behandlung mit Haloperidol, Clonidin oder deren Kombination an einem beliebigen Tag eine Regredienz des Delirs hin zum Folgetag nachzuvollziehen, wohingegen dies bei 2712 Patienten (57,6 %) persistierte. Die übrigen Fälle erreichten eine für die Beurteilung nur unzureichende Wachheit, verstarben, wurden verlegt oder wiesen unvollständige Daten auf. Patienten, die eine Regredienz des Delirs aufwiesen, zeigten in der Analyse der erhaltenen Medikamente signifikant geringere Raten und Dosierungen von Haloperidol (*p* < 0,001), Clonidin (*p* = 0,025) und deren Kombination (*p* < 0,001). Der Effekt war überdies für Haloperidol mit 1 mg innerhalb von 24 h (*p* < 0,0001) und etwas geringer auch für Clonidin mit 0,1 mg innerhalb von 24 h (*p* = 0,023) dosisabhängig.

Die Ergebnisse von Smit et al. [26] überraschen zunächst, weil sie einen inversen statistischen Zusammenhang zwischen der Anwendung von Haloperidol und Clonidin und der Regredienz eines Delirs anhand einer großen Stichprobe zeigen. Von den Autoren selbst wird u. a. angeführt, dass Haloperidol und Clonidin hypoaktive Symptome begünstigen, die mit dem angewandten Delirscreening möglicherweise als falsch-positiv bewertet wurden. Darüber hinaus ist eine symptomorientierte Anwendung von Haloperidol und Clonidin anzunehmen, sodass Patienten mit schwererer deliranter Symptomatik mutmaßlich höhere Dosen erhielten. Naheliegend erscheint daher die Annahme, dass ebendiese Patienten eine geringere Wahrscheinlichkeit für eine Regredienz des Delirs im kurzfristigen Verlauf aufwiesen. Ungeachtet dieser methodischen Limitationen zeigt die Studie von Smit et al. [26] die bei der Detektion des Delirs bestehenden Herausforderungen. Hinsichtlich der evidenzbasierten Behandlung des Delirs sind weiterhin randomisierte kontrollierte Studien notwendig, in denen auch nichtmedikamentöse Maßnahmen und die Delirprävention berücksichtigt werden sollten.

## Zusammenfassung und Ausblick

Auch in den Jahren 2020 und 2021 wurde eine Reihe von Studien veröffentlicht, die den Wissensstand im neurologisch-intensivmedizinischen Bereich substanziell erweiterten (Tab. 1).

**Table 1:** 

Originaltitel der Studie	Ergebnis/Kurzzusammenfassung
Local anesthesia without sedation during thrombectomy for anterior circulation stroke is associated with worse outcome [3]	Eine prospektive, registerbasierte, multizentrische Studie konnte theoretische Vorteile einer Lokalanästhesie als periprozedurales Verfahren bei der mechanischen Thrombektomie gegenüber einer Analgosedierung im Hinblick auf das Behandlungsergebnis nach 3 Monaten nicht bestätigen
Single mean arterial blood pressure drops during stroke thrombectomy under general anaesthesia are associated with poor outcome [12]	In einer retrospektiven, monozentrischen Kohortenstudie war ein periprozeduraler Blutdruckabfall auf einen mittleren arteriellen Druck von weniger als 60 mm Hg mit einem ungünstigen Behandlungsergebnis der mechanischen Thrombektomie nach 3 Monaten assoziiert
Safety and efficacy of intensive blood pressure lowering after successful endovascular therapy in acute ischaemic stroke (BP-TARGET): a multicentre, open-label, randomised controlled trial [17]	In einer randomisierten, multizentrischen Studie zum optimalen systolischen Blutdruckbereich nach einer mechanischen Thrombektomie ergaben sich unter Anwendung der Zielbereiche 100–129 und 130–185 mm Hg keine signifikanten Unterschiede hinsichtlich der Raten an intrazerebralen Blutungskomplikationen und kritischer Hypotensionen
Risk profile of decompressive hemicraniectomy for malignant stroke after revascularization treatment [1]	Eine retrospektive, monozentrische Studie erbrachte für die Behandlungssequenz einer mechanischen Thrombektomie mit nachfolgend notwendig werdender dekompressiver Hemikraniektomie kein vermehrtes Auftreten perioperativer Komplikationen im Vergleich zu operativen Eingriffen, bei denen vorab keine rekanalisierenden Therapien erfolgten
Community-acquired bacterial meningitis in adults: in-hospital prognosis, long-term disability and determinants of outcome in a multicentre prospective cohort [27]	In einer prospektiven, registerbasierten, multizentrischen Studie konnten als häufigster Erreger der ambulant erworbenen bakteriellen Meningitis *Streptococcus pneumoniae* identifiziert und als Risikofaktoren für einen ungünstigen Verlauf u. a. das Patientenalter (> 70 Jahre), fokale neurologische Symptome und eine erst verzögert stattfindende Liquoruntersuchung eingegrenzt werden
Acute ischemic stroke and COVID-19: an analysis of 27,676 patients [21]	Unter Nutzung einer sehr großen Stichprobe lieferte eine retrospektive Untersuchung keinen Unterschied im Auftreten von Schlaganfällen bei Patienten mit COVID-19 gegenüber nichtbestätigten Verdachtsfällen, jedoch waren Schlaganfälle, die im Zusammenhang mit COVID-19 auftreten, mit einer erhöhten Sterblichkeit assoziiert
Haloperidol, clonidine and resolution of delirium in critically ill patients: a prospective cohort study [26]	In einer prospektiven, monozentrischen Kohortenstudie war die Anwendung von Haloperidol und Clonidin im Rahmen einer Delirbehandlung nicht mit einer Regredienz der deliranten Symptomatik im kurzfristigen Verlauf assoziiert

Mehrere Studien fokussierten auf das periprozedurale Management der mechanischen Thrombektomie von proximalen Gefäßverschlüssen. Einzelne Aspekte zur Verfahrenswahl (Analgosedierung vs. Lokalanästhesie) und zum optimalen Blutdruck während und nach der Intervention konnten hierbei adressiert werden, wodurch gleichermaßen der Bedarf weiterer, noch differenzierterer Untersuchungen z. B. zur optimalen Blutdruckobergrenze nach der mechanischen Thrombektomie deutlich wurde. Für die Indikationsstellung der dekompressiven Hemikraniektomie nach einer vorangegangenen medikamentösen bzw. mechanischen Rekanalisationsbehandlung lieferten Beobachtungen zum unmittelbaren perioperativen Risiko mehr Sicherheit bei der individuellen Nutzen-Risiko-Bewertung. Die gelungene Eingrenzung von Faktoren, die mit einem ungünstigen Behandlungsverlauf einer ambulant erworbenen bakteriellen Meningitis einhergehen, kann helfen, diese Patienten frühzeitig zu identifizieren und adäquate therapeutische Maßnahmen einzuleiten. Der Zusammenhang zwischen SARS-CoV‑2 bzw. im Speziellen COVID-19 und ischämischen Schlaganfällen bleibt vielschichtig. Zu bestätigen scheint sich die Beobachtung eines potenziell komplikationsbehafteten Verlaufs im Falle eines unter COVID-19 eintretenden Schlaganfalls. Die optimale Behandlung des Delirs bleibt eine Herausforderung, und in diesem Zusammenhang ergeben sich auch grundsätzliche Fragen, wie beispielsweise das optimale Screening und die Verlaufsbeurteilung der Erkrankung sowie der Einfluss nichtmedikamentöser Maßnahmen.
